# Multi-walled carbon nanotubes functionalized with recombinant *Dengue virus 3* envelope proteins induce significant and specific immune responses in mice

**DOI:** 10.1186/s12951-017-0259-4

**Published:** 2017-04-04

**Authors:** Alice F. Versiani, Ruiz G. Astigarraga, Eliseu S. O. Rocha, Ana Paula M. Barboza, Erna G. Kroon, Milene A. Rachid, Daniele G. Souza, Luiz O. Ladeira, Edel F. Barbosa-Stancioli, Ado Jorio, Flávio G. Da Fonseca

**Affiliations:** 1 Laboratory of Basic and Applied Virology, Departamento de Microbiologia, Instituto de Ciências Biológicas, Universidade Federal de Minas Gerais, Belo Horizonte, MG Brazil; 2 Laboratório de Vírus, Departamento de Microbiologia, Instituto de Ciências Biológicas, Universidade Federal de Minas Gerais, Belo Horizonte, MG Brazil; 3 Departamento de Patologia, Instituto de Ciências Biológicas, Universidade Federal de Minas Gerais, Belo Horizonte, MG Brazil; 4 Departamento de Física, Instituto de Ciências Exatas, Universidade Federal de Minas Gerais, Belo Horizonte, MG Brazil; 5 Laboratório de Nanoscopia, Departamento de Física, Universidade Federal de Minas Gerais, Belo Horizonte, MG Brazil; 6 Laboratory of Microorganism-Host Interaction, Departamento de Microbiologia, Instituto de Ciências Biológicas, Universidade Federal de Minas Gerais, Belo Horizonte, MG Brazil

**Keywords:** Dengue vaccine, Carbon nanotubes, Subunit vaccine, Nanoconjugate

## Abstract

**Background:**

Dengue is the most prevalent arthropod-borne viral disease in the world. In this article we present results on the development, characterization and immunogenic evaluation of an alternative vaccine candidate against Dengue.

**Methods:**

The MWNT-DENV3E nanoconjugate was developed by covalent functionalization of carboxylated multi-walled carbon nanotubes (MWNT) with recombinant dengue envelope (DENV3E) proteins. The recombinant antigens were bound to the MWNT using a diimide-activated amidation process and the immunogen was characterized by TEM, AFM and Raman Spectroscopy. Furthermore, the immunogenicity of this vaccine candidate was evaluated in a murine model.

**Results:**

Immunization with MWNT-DENV3E induced comparable IgG responses in relation to the immunization with non-conjugated proteins; however, the inoculation of the nanoconjugate into mice generated higher titers of neutralizing antibodies. Cell-mediated responses were also evaluated, and higher dengue-specific splenocyte proliferation was observed in cell cultures derived from mice immunized with MWNT-DENV3E when compared to animals immunized with the non-conjugated DENV3E.

**Conclusions:**

Despite the recent licensure of the CYD-TDV dengue vaccine in some countries, results from the vaccine’s phase III trial have cast doubts about its overall efficacy and global applicability. While questions about the effectiveness of the CYD-TDV vaccine still lingers, it is wise to keep at hand an array of vaccine candidates, including alternative non-classical approaches like the one presented here.

## Background

Dengue is currently the most prevalent arthropod-borne viral disease in the world [1]. The exact global burden of the disease is difficult to pinpoint, as epidemiological surveillance in many affected countries is poor and under-notification is significant. Still, conservative and non-conservative projections have put the disease numbers in around 390 million cases per year, of which one-fourth demand medical attention [2]. The arrival of an effective vaccine against *Dengue virus* will certainly modify this picture. In this context, the recent licensure of the Sanofi Pasteur’s Dengvaxia^®^ (CYD-TDV) vaccine represents a milestone in the efforts to control the disease. Pooled results from two phase III trials, conducted in Asia and Latin America, revealed that the CYD-TDV vaccine presents an overall efficacy of 59.2%, and that could reach up to 65.6% when data from individuals under age of 9 are excluded. Therefore, the vaccine’s current licensure comprehends only individuals that are 9 years-old or older [3]. These results have been considered somewhat disappointing by many experts in the field, highlighting the strategic importance to keep on going studies on alternative dengue vaccines.

Most dengue vaccines being tested in pre-clinical or clinical settings include the presence of the *Dengue virus* (DENV) envelope protein in their formulations. The DENV envelope protein (E) is an immunodominant polypeptide that is exposed on the virus surface and encompasses functions that include adsorption to host cells and membrane fusion during the penetration phase of the virus life cycle [4]. The E protein is primarily produced as part of a polyprotein during infection, and as the cycle progresses it is cleaved off the polyprotein by a virus-coded protease [5]. These features are shared by all members of the *Flavivirus* genus within the *Flaviviridae* family—a group of ssRNA(+) viruses to which DENV belongs. There are four genetically and antigenically distinct DENV serotypes (DENV1-4), and each one is able to cause a full range of clinical manifestations that go from asymptomatic infections to severe hemorrhage and organ failure [6, 7]. Because there are few immunogenic motifs in the E protein that are conserved among all serotypes, most dengue immunogens have to include components from each individual serotype in the final vaccine formulation.

Nanomaterials have a broad spectrum of applications in the bioengineering and pharmaceutical fields [8], and carbon nanotubes (CNT) [9] are among the most versatile and well characterized members of this group of materials. CNTs are tubular hollow structures with the walls formed by one-atom-thick sheets of sp^2^ bonded carbon [8]. These nanostructures can be found in two classes: single walled carbon nanotubes (SWNT), which are formed by a single cylindrical graphene layer; and multi-walled carbon nanotubes (MWNT) comprising several concentric layers of graphene. Functionalized carbon nanotubes (ƒ-CNT) have been considered as a promising carrier for antigen and drug delivery due to many important characteristics that include excellent biocompatibility when properly treated and the ability to penetrate through the cell membranes by passive diffusion [10, 11]. Carbon nanotubes have been tested as antigen carrier in several vaccine studies, including trials against infectious diseases [12–15] and cancer [16–18].

Our group has demonstrated the ƒ-CNT potential as an antigen carrier against the intracellular bacteria *Anaplasma marginale* [19] and, more recently, we demonstrated the potential of ƒ-CNT to carry a tetravalent plasmid-based DNA vaccine against dengue [20]. In the later study, although transcription of the plasmid elements were not increased by the DNA association to CNTs, an augmentation in the levels of specific antibody-producing B cells were observed upon mice immunization when compared to mice vaccinated with naked plasmids. Nonetheless, there has been considerable criticism towards the use of DNA-based vaccines as all human trials exploring the approach have failed to deliver same levels of immunogenicity seen in pre-clinical studies using small mammals [21]. Thus, as an alternative, we took advantage of the ƒ-CNT potential as an antigen delivery agent to design and test a protein-based immunogen against dengue.

## Results

### Characterization of the MWNT-DENV3E immunogen

**Fig. 1:**
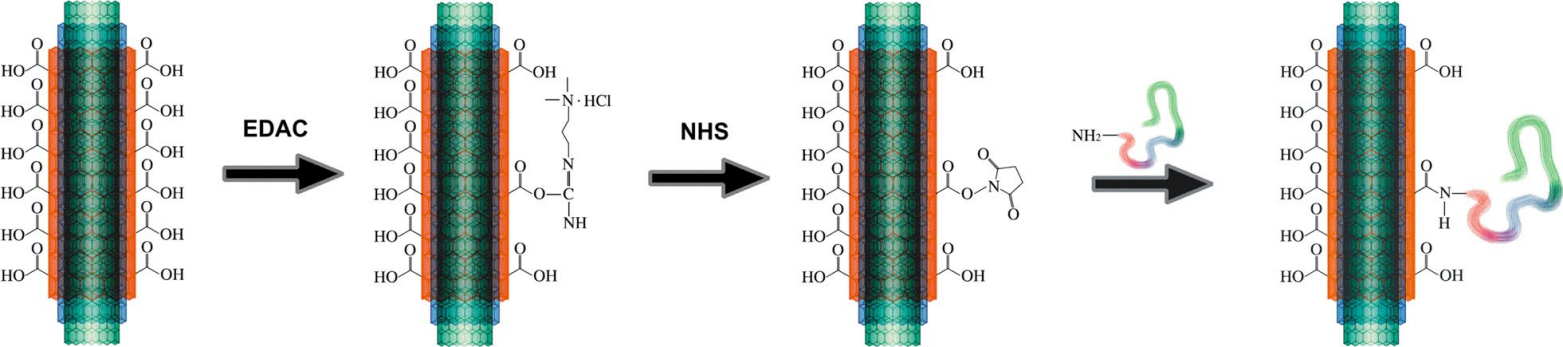
Generation of DENV3E-functionalized MWNT (MWNT-DENV3E). The *scheme* depicts the two-step process of diimide-activated amidation and the covalent attachment of DENV3E proteins to the carbon nanotubes’ surface. *EDAC* 1-Ethyl-3-(3-dimethylaminopropyl)carbodiimide; *NHS N*-hydroxysuccinimide. The DENV3E protein is represented by the *colorful wavy line*. Diagram is not to scale

**Fig. 2:**
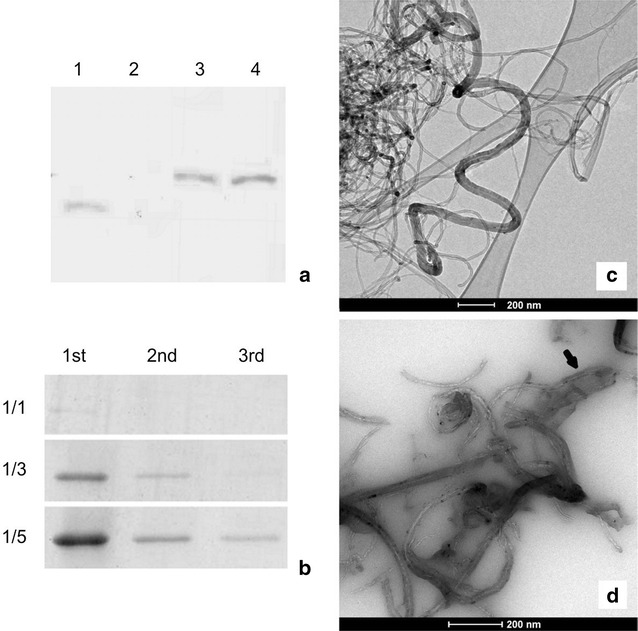
Characterization of the DENV3E recombinant protein and the MWNT functionalization process. **a** Western blot analysis:* column 1* was loaded with an irrelevant recombinant protein (37 KDa) and probed with its respective specific antibody as an external positive control.* Column 2* was loaded with an eluate from non-transformed bacteria, as negative control, and probed with monoclonal anti-DENV antibodies.* Columns 3* and* 4* were loaded with eluates from recombinant DENV3E (~50 kDa) producing bacteria and probed with either monoclonal anti-DENV antibodies or polyclonal anti-HIS antibodies, respectively. **b** After functionalization, the MWNT-DENV3E solution was washed 3 times (1st, 2nd and 3rd) to remove unbound proteins and the flow through was analyzed by SDS-PAGE. Three different MWNT to protein ratios (w/w using 1 µg of MWNT) were tested (1/1, 1/3 and 1/5). **c** TEM of carboxylated MWNT. **d** TEM of MWNT-DENV3E, *black arrow* points to typical deposition of amorphous material on the protein-functionalized MWNT surface. *Scale bars* are represented in both **c**, **d**

We designed an anti-dengue—serotype 3—immunogen based on the covalent binding of recombinant DENV3E to carboxylated MWNTs (Fig. 1). The antigenic potential of the DENV3E recombinant protein (of approx. 50 kDa) used in our constructs was initially validated by an immunoblot using monoclonal antibodies against *Dengue virus* (Abcam^®^, USA) (Fig. 2a). Identical blots were also probed with an antibody against the histidine tail epitope (Fig. 2a) as the pQE9 expression plasmid drives the addition of a 6xHis-tag to the produced protein.

The efficiency of the MWNT functionalization with DENV3E was confirmed by two independent approaches. First, after the attachment of proteins, the reaction solution was centrifuged and the flow through was tested by SDS-PAGE in order to evaluate which MWNT to protein w/w ratio would generate maximum functionalization with minimal unbound protein. We determined that the 1/3 ratio achieved maximum MWNT saturation with minimal protein loss (Fig. 2b). Second, microscopy and spectroscopy analysis were used to elucidate structural aspects of the constructs. Both carboxylated and protein-functionalized MWNTs were examined by transmission electron microscopy (TEM) and atomic force microscopy (AFM). After synthesis, MWNT were somewhat heterogeneous, with diameters that ranged from 20 to 40 nm and lengths ranging from 40 to 60 μm. Before sonication, carboxylated nanotubes tended to aggregate quickly, but after sonication MWNT were soluble in water for periods of up to 2 months at 4 °C. No other experiments concerning stability of the nanoconjugates were conducted. Figure 2c shows typical MWNTs agglomerated upon evaporation of the solvent seen through TEM. Figure 2d shows a similar TEM preparation containing MWNT-DENV3E. Areas of amorphous material around the nanotubes can be observed, and these probably correspond to regions where the recombinant protein is attached to the surface of the MWNTs (thick arrow). Such areas are absent in non-functionalized MWNT preparations.

**Fig. 3:**
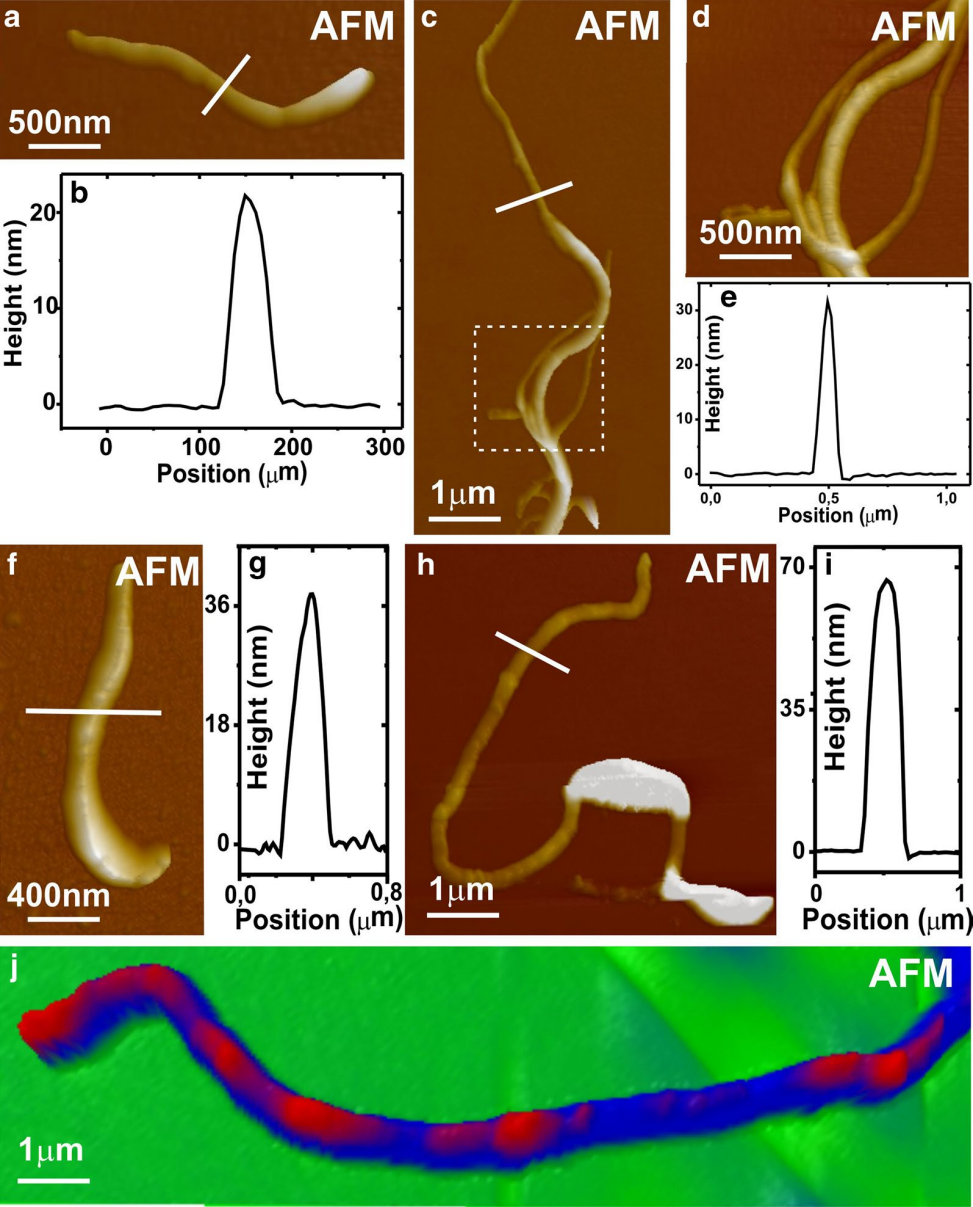
Atomic force microscopy of non-functionalized MWNT and MWNT-DENV3E samples. **a**, **c** represent AFM images of different MWNTs before functionalization with the DENV3E protein. Measurements of the typical MWNT height (width) in different samples are shown on **b**, **e**. **d** is an enlarged image of the area marked with a *doted square* in **c**. **f** and **h** are AFM images of different DENV3E-functionalized MWNT samples. Their respective height (width) measurements are shown on graphs **g**, **i**. **j** depicts an enlarged image of the tube showed in **h**, and rings of deposited material thought to be the recombinant protein (in *red*) can be seen on the surface of the tube (in *blue*). *Scale bars* are represented

AFM analyses were conducted to confirm and complement the observations made by TEM. Figure 3a–d show carboxylated MWNTs before functionalization with the recombinant protein. The average height on the sections (width of the nanotube), is around 20 nm (Fig. 3b, e). On the other hand, images of the nanotubes after functionalization with DENV3E show a significant increase in nanotube width due to the binding of the recombinant protein (Fig. 3f–j). Figure 3j represents an enlarged image of the nanotube depicted in Fig. 3h, where regions presenting increased surface height thought to be protein rings (in red) can be observed on top of the MWNT (in blue).

**Fig. 4:**
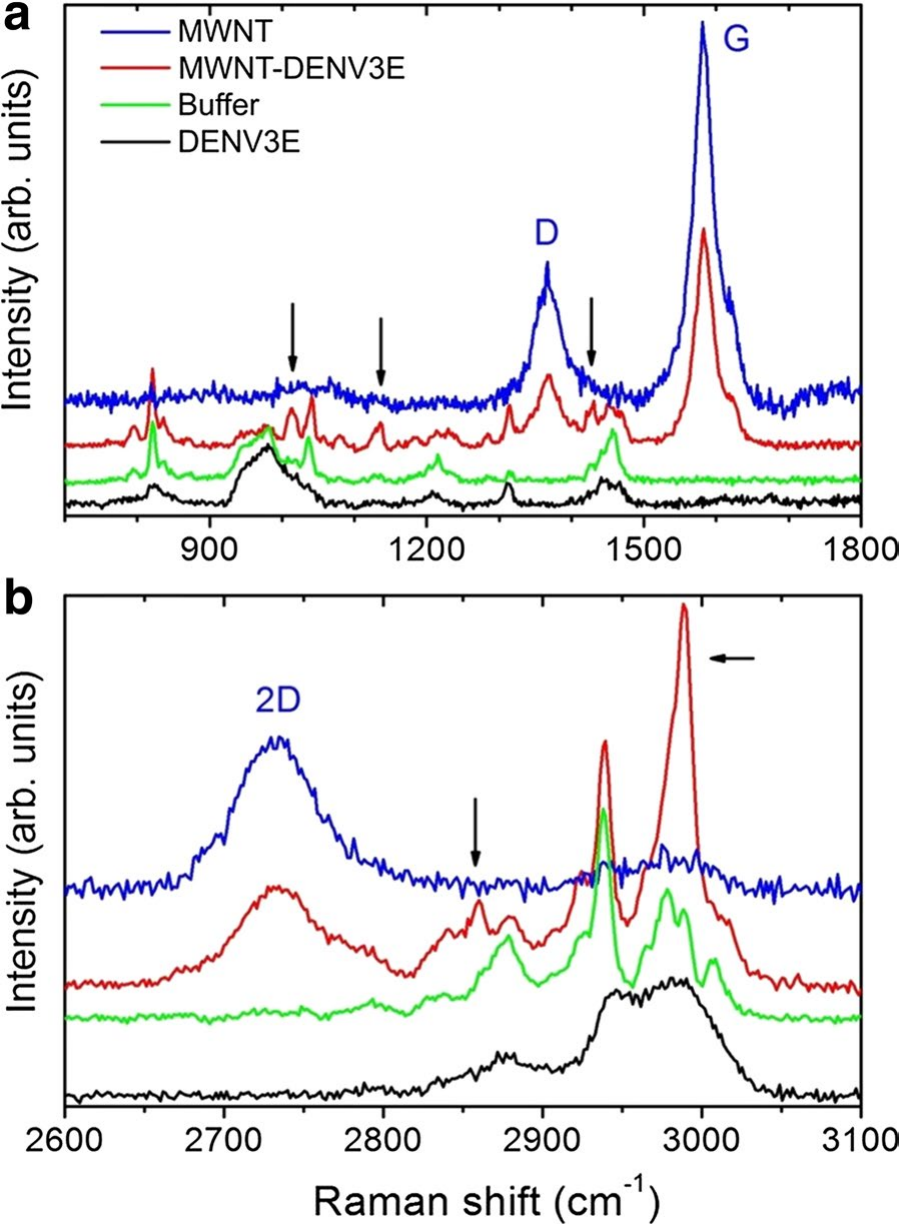
Raman spectroscopy of carboxylated MWNT and MWNT-DENV3E samples. **a**, **b** in two different spectral regions, the Raman signatures for the samples: DENV3E protein in *black*, buffer solution in *green*, DENV3E + nanotube mixture in *red*, and the pure carbon nanotubes in *blue*. The spectrum for the DENV3E + nanotube sample clearly demonstrates the presence of the two agents

To confirm the microscopy-based structural observations, the MWNT-DENV3E immunogen was analyzed by Raman spectroscopy. In Fig. 4a it is possible to note the bands in 821, 980 and 1450 cm^−1^, attributed to the spectroscopic signature of the buffer solution. These bands also appear in the DENV3E and in the DENV3E + nanotube mixture, since the buffer solution is present in these samples as well. The observation of the disorder induced mode (D band, near 1367 cm^−1^) in the Raman spectrum of the pure nanotube sample is related to the carboxylation procedure that creates defect centers for enhancing the nanotube chemical activity. The G band at 1583 cm^−1^ is attributed to C–C stretching mode. The ratio between the intensities of D and G bands is used as a measure of disorder, and in nanotube sample it is 0.33, while in the DENV3E + nanotubes it was found to be 0.39. According with previous characterization protocols [22], this is a minor change indicating that the functionalization procedure does not degrade the carbon nanotubes significantly, as expected based on the model that an amide bonds on a previously created carboxyl group, so that new defects are not created in the CNT wall. Figure 4b shows the spectral region where the 2D overtone (2730 cm^−1^) of the nanotube is observed. The small broad band at 2980 cm^−1^ is also associated with the carboxylation procedure. The DENV3E protein broad features noted in 2800 and 3500 cm^−1^ are vibrational modes related to stretching of C–H bonds in different configurations, as well as other peaks highlighted by the black arrows in Fig. 4a, indicating configurational changes of the protein in the mixture sample.

### Evaluation of humoral responses in immunized mice

**Fig. 5:**
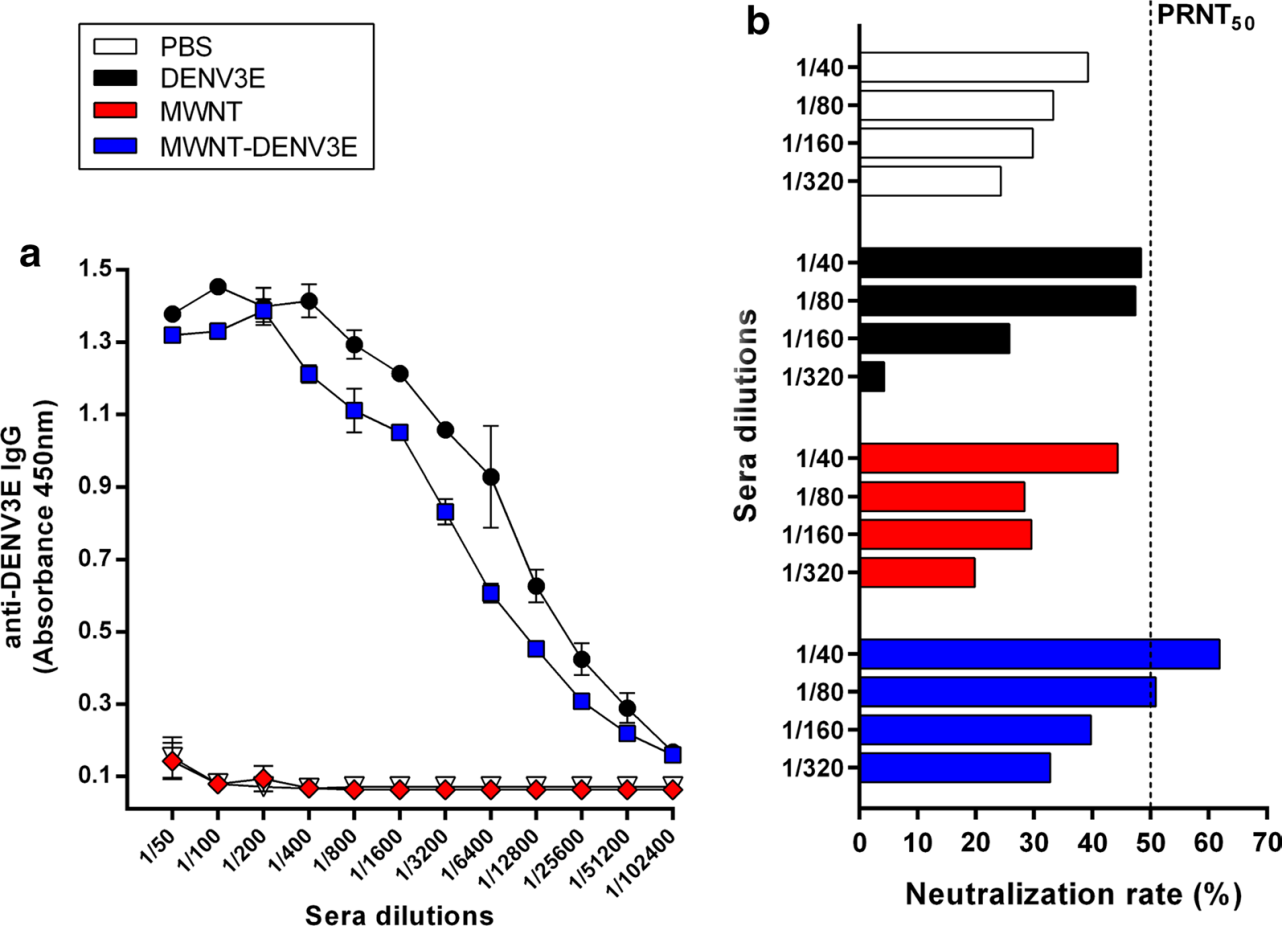
Induction of specific anti-DENV3 humoral responses in immunized mice. Animals were immunized with either MWNT-DENV3E, MWNT, DENV3E or PBS plus adjuvant using a prime-boos-boost regimen. 14 days after the last boost animals were bled and the generation of anti-DNEV3 was assayed. **a** Immunization with MWNT-DENV3E and DENV3E induced similarly high anti-DENV3E IgG responses, with detectable titers of up to the 1/51200 serum dilution. **b** Mice immunized with MWNT-DENV3E showed the higher PRNT_50_ titers (1/80), while groups immunized with DENV3E, MWNT and saline showed only background levels of 1/20. *Dotted line* represents the 50% neutralization rate cut-off

Having determined that the MWNT-DENV3E nanoconjugate was correctly obtained, immunization protocols were designed to evaluate its immunogenicity. Groups of mice were inoculated with MWNT-DENV3E, MWNT, DENV3E or PBS in a prime-boost-boost regimen. Other than a visible inflammation spot on the site of injection, animals immunized with any of the antigens showed neither signs of disease nor significant weight loss during the evaluated period. Anti-DENV3 antibody responses elicited upon immunization with the different immunogens were evaluated in the pooled sera from each group by an IgG-ELISA. Results show that mice immunized with MWNT-DENV3E and DENV3E induced specific anti-DENV3E antibody responses (Fig. 5a). As expected, mice that received saline solution or non-functionalized MWNT did not show any detectable specific antibody production.

Production of specific neutralizing antibodies by the host is regarded as a requirement to effectively block DENV infection or reinfections [23]. Thus, we looked at the ability of the MWNT-DENV3E immunogen to induce the production of neutralizing antibodies in comparison to mice immunized with the recombinant protein alone. Results revealed background PRNT_50_ titers of 1/20 or less in the groups inoculated with PBS and MWNT. A similar low titer was also obtained when animals were inoculated with the DENV3E soluble protein. Importantly, mice immunized with MWNT-DENV3E showed a significantly higher specific neutralizing antibody titer of 1/80 (Fig. 5b), evidencing that the association of the recombinant protein to MWNTs was sufficient to boost the antigenic potential of DENV3E.

### Cell-mediated responses evaluation

**Fig. 6:**
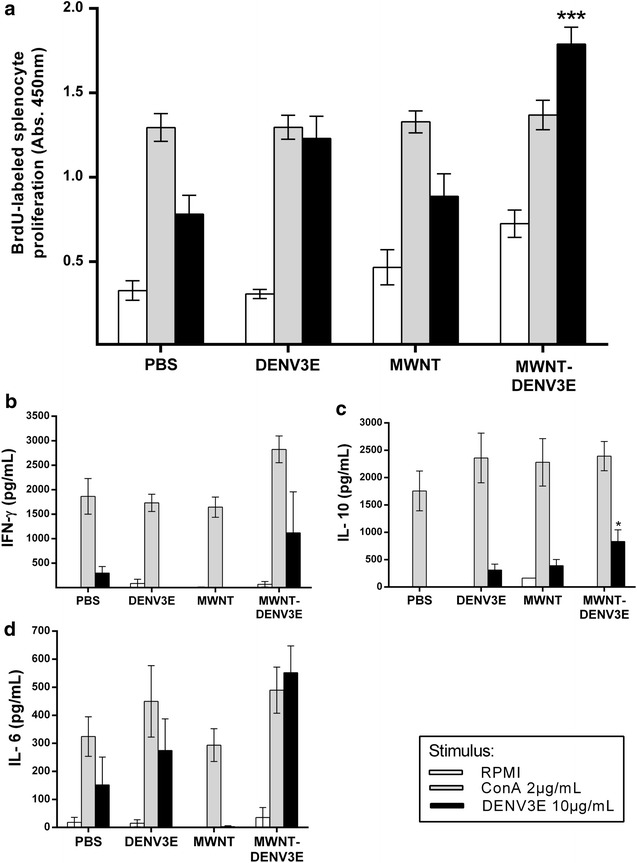
Induction of specific anti-DENV3 cell-mediated responses in immunized mice. **a** Lymphocyte proliferation for each group of immunized mice, after stimulation with recombinant DENV3 (*black bars*), RPMI (*white bars*, negative controls) and ConA (*grey bars*, positive controls). After DENV3E stimulation, cells from mice immunized with MWNT-DENV3E showed a significantly higher level of proliferation compared to cells from mice that received saline. The same result was not observed for cells from mice immunized with non-conjugated DENV3E and MWNT, being statistically similar to mice that received saline. The RPMI and ConA controls behaved as expected. **b**–**d** Respectively, IFN-γ, IL-6 and IL-10 induction levels were evaluated on the supernatant of cultured splenocytes after DENV3E stimulus. The production of these cytokines appear higher in cells from animals immunized with MWNT-DENV3E, when compared to the other groups. Statistical significance was achieved only when IL-10 was assayed in MWNT-DENV3E groups stimulated with the recombinant DENV3E protein (statistical test: one-way ANOVA with Dunn’s multiple comparisons test ***P≤0.001; *P≤0.05)

Splenocytes from immunized and non-immunized mice were stimulated, ex vivo, with purified recombinant DENV3E. The proliferation of cultured cells was measured through BrdU incorporation into genomes of dividing cells. As expected, ConA-stimulated controls (Fig. 6a, grey bars) showed constant high levels of splenocyte proliferation in all mice groups, whereas the RPMI-stimulated controls (Fig. 6a, white bars) showed low background levels. Importantly, there were no statistically significant variations amongst these controls. Among the DENV3E-stimulated cells (Fig. 6a, black bars), on the other hand, only the group immunized with MWNT-DENV3E showed significantly higher levels of splenocyte proliferation in comparison to negative controls. These results—together with those obtained for the specific antibody responses—suggest that the association of the recombinant protein to carbon nanotubes is able to render the DENV3E antigen more immunogenic than the use of the recombinant protein alone.

To further analyze the ability of the MWNT-DENV3E to stimulate cell mediated immune responses, we looked for cytokine production and secretion in PBMCs from immunized mice after DENV3E stimulus. The production of IFN-γ, IL-6 and IL-10 was apparently higher in cells from MWNT-DENV3E-immunized animals when compared to the other groups (Fig. 6b–d). Nonetheless, statistical significance among all groups was achieved only when IL-10 was assayed (Fig. 6d).

## Discussion

The emblematic licensure of the CYD-TDV vaccine in some countries after the conclusion of two large multicenter phase III trials represents a milestone in the global efforts to control Dengue. Nevertheless, despite high expectations, efficacy and risk assessments limited the vaccine’s use to those above 9 years of age and <60 years (45 y.o. in some countries). Variations in relation to specific DENV serotypes were also relevant, as efficacy in the pooled results from the two major trials ranged from 47.1 (against DENV2) to 83.2% (against DENV4) in those above 9 years old [3]. Moreover, a recent mathematical modeling employing data from the trials corroborated previous studies suggesting that the vaccine would have a positive impact in recipients who had been previously infected with DENV, but could have a deleterious effect in patients who were seronegative at the time of vaccination [24]. Those uncertainties have led the WHO to recommend vaccination only in places where seroprevalence is above a 50% threshold [25]. As debates over the applicability of the CYD-TDV persist, it is consensual and prudent to maintain a diverse pipeline of DENV vaccine candidates. In this article we have presented results on the development, characterization and analyses of one such vaccine candidate composed of MWNTs covalently functionalized with a recombinant DENV3E protein produced in *E. coli*.

We have recently evaluated the potential of CNTs to deliver a DNA-based vaccine against Dengue [20]. In that study, the association of the vaccine plasmid to CNTs increased the amount of B-cells producing antibodies in comparison to the naked plasmid. Nevertheless, repeated failures on the use of DNA-based vaccines in human trials prompted us to design and test a more conservative immunogen composed of the DENV envelope protein (serotype 3) associated to MWNTs.

**Fig. 7:**
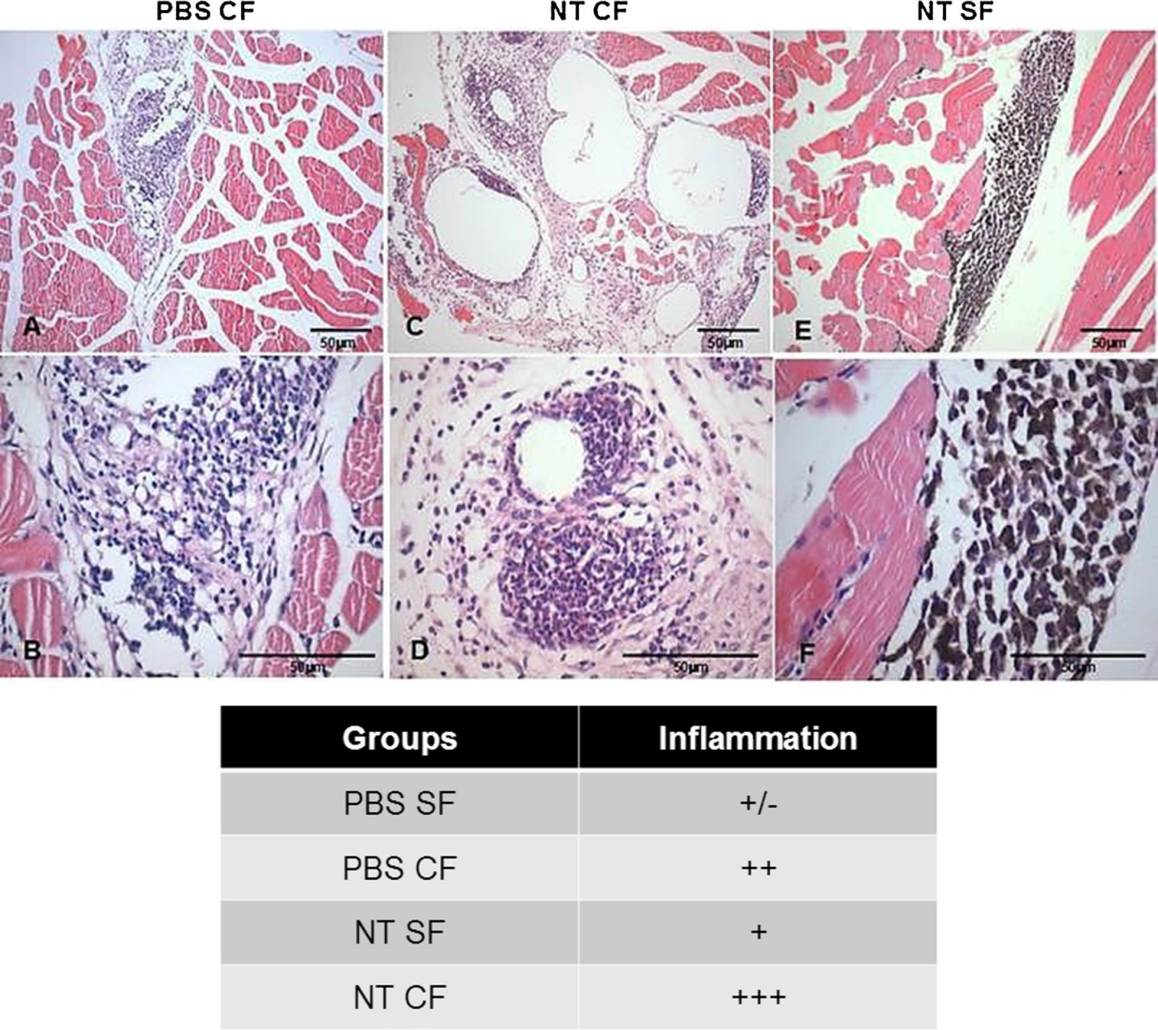
Histopathological findings in animals inoculated with MWNT-DENV3E in the presence or absence of adjuvant. 10 weeks old female Balb/c mice were inoculated with the nanoconjugate in the presence or absence of freund complete adjuvant. Seven days after inoculation fragments of dermal and muscular tissues surrounding the injection sites were fixed and prepared for histopathological analyses using hematoxylin and eosin dies. Animals inoculated with PBS or PBS plus adjuvant were used as controls. **A**, **B** are representative of animals inoculated with PBS plus adjuvant (*PBS CF*); **C**, **D** are representative of animals inoculated with MWNT-DENV3E in the presence of adjuvant (*NT CF*); and **E**, **F** are representative of animals inoculated with MWNT-DENV3E in the absence of adjuvant (*NT SF*). *Upper* and *lower panels* are figures with different magnifications. *Scale bars* are included. The *table* represents an interpretation of the histopathological panels, where − is absence of alterations; + indicates light alterations; ++ indicate moderate alterations; and +++ indicate intense alterations. Figures from animals inoculated with PBS only (*PBS SF*) are not shown

The use of CNTs as delivery agents has met considerable resistance and most criticism comes from high CNT toxicity when these compounds are absorbed through the respiratory tract of humans and animals. To look into that, we performed histopatological analyses on animals inoculated with nanoconjugates in the presence or absence of adjuvants. Our results indicate that nanoconjugates inoculated in the absence of adjuvant induced very light inflammation in the muscular and skin tissues surrounding the site of injection (Fig. 7E–F). Neutrophils and macrophages are visible, and many macrophages presented the accumulation of dark pigments in their interior, thought to be nanoconjugates. When the MWNT-DENV3E antigen was inoculated in the presence of adjuvant, on the other hand, an extensive inflammatory infiltrate was found in the injection site, including the presence of neutrophils, macrophages, lymphocytes and other inflammatory cells (Fig. 7C–D). Nonetheless, important infiltrations of neutrophils, macrophages and lymphocytes are also seen when the adjuvant was inoculated alone (Fig. 7A–B), indicating that at least part of the observed effect is due to the inflammatory properties of the adjuvant, and not by the nanoconjugate itself. No alterations were seen when animals were inoculated with PBS only (not show). When present, nanoconjugates apparently accumulated in the subdermal site of injection; however, no further studies on the biodistribution of MWNT-DENV3E were conducted. Nonetheless, whether CNTs are inherently cytotoxic at any organic systems has yet to be definitely proven. Simon et al. [26] tested levels of MWNT cytotoxicity in different eukaryotic cell lines and concluded that exposition to the compound rendered neither cytotoxicity nor endocrine disruption to any of them. Furthermore, it is widely accepted that functionalization of CNTs reduces their toxicity and also increases biocompatibility [27]. It has also been shown that CNTs are able to activate immune cells without causing any cytotoxicity if appropriately functionalized [28]. In our study, the correct functionalization of MWNTs with a purified recombinant DENV3E protein was tested by complementary methods (Raman spectroscopy, TEM and AFM). In all tests, the successful association between the MWNTs and the target protein was implied. As for the choice for the envelope protein as the immunogenic antigen, it has been long established that the E protein—or at least epitopes originated from it—is an obligatory component of any vaccine strategy against dengue, either alone or in combination with other viral antigens [29, 30].

The potential of MWNT as a delivery agent on a dengue vaccine candidate became obvious when we analyzed the immunogenicity of the MWNT-DENV3E in vivo, especially after comparing the results with those obtained upon immunization with the non-conjugated DENV3E protein. The inefficiency of anti-dengue subunit vaccines using recombinant proteins in adjuvant containing formulas is largely recognized. There are exceptions, as is the case of a tetravalent subunit vaccine composed of the E protein produced on a *Drosophila* S2 cell expression system [31]. Nonetheless, most subunit vaccines against dengue have been disregarded as candidates worth going to human trials [32]. Part of the failure of most anti-dengue subunit vaccines has been attributed to the poor generation of DENV specific neutralizing antibodies in immunized individuals [33]. Not surprisingly, the non-conjugated DENV3E protein used in our study failed to induce titers of neutralizing antibodies above the negative control thresholds, although titers of broad anti-DENV3E antibodies were detected by ELISA (Fig. 5a). Strikingly, while inducing comparable amounts of broad anti-DENV3E antibodies in relation to the non-conjugated DENV3E protein, the inoculation of MWNT-DENV3E generated higher titers of anti-DENV3E neutralizing antibodies (Fig. 5b). One hypothesis to explain how the association of MWNT has so dramatically changed the immunogenic potential of the very same protein is that the functionalization of the DENV3E with MWNT may have led to a differential profile of antigenic presentation. Moreover, it has been shown that ƒ-CNTs have immunostimulatory properties and are able to induce the production of cytokines [26, 34]. Thus, the increased neutralizing antibody titers could have been a result of CNT-driven immunomodulation.

Another crucial aspect that may have hampered the use of subunit vaccines against DENV infections is their general lack of robust cell responses upon immunization. Despite the possible participation of T cells in the immunopathogenesis of DENV infections and severe dengue [35], their importance as a requirement for an effective anti-DENV vaccine is being considered [36]. Similarly to what we observed for the generation of humoral responses, cell responses were statistically higher in splenocytes derived from mice immunized with MWNT-DENV3E when compared to animals inoculated with non-conjugated DENV3E (Fig. 6a). Likewise, cytokine levels measured in the splenocyte culture supernatants revealed the same tendency in which association of DENV3E to the CNT carrier led to an apparent augmentation of cytokine production, although only the increase in IL-10 production was statistically superior in animals immunized with the nanoconjugate.

The immune response to a specific antigen depends on its delivery through the adequate pathway in order to generate the desired outcome [37]. Inert antigens, such as the DENV3E immunogen showed here are mostly dependent on protein up taking by professional APCs so they can be presented to the vaccinee’s adaptive immune system. This way, the antigen is mostly presented through association with MHC-II molecules, leading to a predominantly Th2 type of response. CNTs (MWNTs) present the inherent ability to passively penetrate through cell membranes, thus performing intracellular delivery of any biomolecule that might be associated with them [10, 11, 38]. Because internalization of the CNT-associated antigens is not dependent on APC phagocytosis, such antigens could be presented by cells other than APCs (and APCs as well), leading to peptide association with MHC-I molecules and a more broad Th1/Th2 immune response.

Although reactogenic and no longer used as adjuvant in currently available vaccines, we chose to employ Freund’s adjuvant in our formulation in order to exploit its known ability to induce strong T cell responses. The wall components of inactivated mycobacteria (present in the complete form of the Freund’s adjuvant) are potent inducers of T cell-mediated responses and are known to activate human TLR2 and TLR4 [39]. Isolated components of mycobacteria cell wall have been proven less reactogenic than full cell extracts while retaining important Th1-biased immune-stimulatory properties [39, 40]. Experimental vaccines using such components have shown promising results against a variety of infectious diseases [41, 42], including dengue [43]. In our study, all groups—including the PBS control group—had the same adjuvant included, indicating that differences observed were not due to the choice of adjuvant, but to the intrinsic properties of the MWNT-DENV3E immunogen. Still, the further development of this immunogen will require the use of a less reactogenic adjuvant or even no adjuvant at all.

## Conclusions

Here we have shown that, when used as a delivery tool, CNTs were able to significantly boost the antigenicity of an otherwise poorly immunogenic antigen. Indeed, the association of CNTs to the same immunogen has increased substantially the generation of both cell-specific and neutralization antibody responses against bacteria-made DENV3 envelope proteins. Subunit and inactivated vaccines are considered safer than live vaccines because the first do not include replication competent components in their formula, which is usually associated with excessive reactogenicity. On the other hand, inert antigens are more unstable upon vaccination and also less likely to induce robust cell-mediated responses [44]. Improving both stability and immunogenicity of safer antigens is a key aspect in the quest for better vaccines. The experimental antigen tested here generated responses that would be specific for *Dengue virus* serotype 3 only, but the presented results generated good perspectives for the testing of proteins derived from the other dengue serotypes. The generation of an alternative, non-live anti-dengue vaccine candidate with good perspectives, as the one presented here, may add important options to a rather limited and difficult scenario, especially if we consider the increasing gaps on the immunogenic coverage presented by the currently licensed dengue vaccine.

## Methods

### Recombinant DENV3E protein expression and purification

The recombinant E protein from DENV3 (DENV3E) was expressed in *E. coli* using a virus isolated from a patient in Brazil as the DENV gene source [44]. A pQE9 plasmid carrying the DENV3E gene was used and the construction details are described elsewhere [45]. The cloned gene codes for the 80% N-terminal portion of the DENV3E, amino acids 1 to 404, as the removal of the protein hydrophobic C-terminus has been shown to increase protein yield during its heterologous expression as well as its immunogenicity [46].

For protein purification, DENV3E-expressing *E. coli* cells were disrupted using 6M guanidine buffer under agitation and then homogenized (French Press Cell Disrupter, Thermo Electron Corporation). After centrifugation at 7080*g* for 1 h, the supernatant was collected and the recombinant protein was purified by nickel affinity chromatography under denaturing conditions. Ni-NTA Agarose resin (Qiagen, USA) was added to the supernatant and the solution was incubated for 2 h under constant agitation to allow binding between the resin and 6xHis-tagged proteins. After centrifugation, the pellet was suspended in 8M urea buffer (pH 8) and the resin-bound protein solution was added to the chromatography column. Fractions were screened by SDS-PAGE, quantified by Bradford (BioRad, USA) and a pool of the most productive samples were concentrated using VivaSpin (GE Healthcare, USA) with a 30 kDa membrane cutoff.

### MWNT-DENV3E functionalization

For the development of the nanoconjugate, fragmented and carboxylated Multi-Walled Carbon Nanotubes (MWNT) [47] were covalently functionalized to the recombinant DENV3E protein through diimide-activated amidation [48]. The method is based on the covalent binding of the protein amine portions to the carboxylic groups on the MWNT surface through the addition of the coupling agent 1-ethyl-3-(3-dimethylaminopropyl)-carbodiimide (EDAC). The reaction is stabilized by adding *N*-hydroxysuccinimide (NHS) in MES buffer (Fig. 1). After functionalization, the MWNT-DENV3E was centrifuged to remove chemical residues and free proteins and then suspended in MES buffer. The functionalization efficiency was determined by testing three different w/w MWNTs to DENV3E ratios (using 1 µg of MWNT): 1/1, 1/3 and 1/5. After washings, the flow through was analyzed by SDS-PAGE in order to detect unbound proteins and determine the MWNT saturation.

### TEM

The size and morphology of carboxylated MWNT and MWNT-DENV3E were evaluated by transmission electron microscopy (TEM) using a 120 kV FEI Technai G2-12 (Spirit BioTwin) microscope. Materials were deposited onto carbon grids (PELCO^®^) by dripping method without any further processing.

### AFM and Raman spectroscopy

For atomic force microscopy (AFM) and Raman spectroscopy analyses, carboxylated MWNT or MWNT-DENV3E were deposited onto a 300 nm-thick SiOx layer on top of a silicon substrate using a dripping method—dry nitrogen were used to prevent material agglomeration without any further processing. AFM images were acquired using a scanning probe microscopy (SPM) (XE-70 from Park Instruments) operating on intermittent contact mode. Silicon cantilevers (PPP-NCHR) from Nanosensors with nominal spring constant k~42 N m^−1^, nominal radius of curvature R~10 nm and resonant frequency ω0 ~ 330 kHz were used. The Raman spectroscopy was performed with a Renishaw Invia Reflex spectrometer in the single-mode configuration. The spectrometer resolution was 2 cm^−1^ using the 1800 l mm^−1^ grating and a 50× objective. The power used in the measurements was of 2 mW at the objective. The excitation line was of 457 nm, provided by a Coherent Innova 70C Spectrum Ar+/Kr+ laser. All measurements were performed with 1 acquisition of 100 s.

### Immunization protocol

To evaluate the immunogenicity of MWNT-DENV3E, 10 weeks old healthy female Balb/c mice were divided into four groups and immunized subcutaneously with 200 µL of solutions containing either MWNT-DENV3E (25 µg of protein), MWNT, DENV3E (25 µg of protein) or PBS, using the same three-dose protocol (prime-boost-boost). Initial doses were mixed with Freund’s complete adjuvant and further boosting doses were prepared with Freund’s incomplete adjuvant. Doses were administered on 21 days intervals. After 14 days past the last boost, animals received 120 µL of anesthetics (ketamine 21% xylazine 8% in saline buffer) and were bled. The anesthetized mice were euthanized and spleens were immediately harvested. Fragments of dermal and muscular tissues surrounding the inoculation sites of nanoconjugates in the presence or absence of adjuvant were fixed and prepared for histopathological analyses using hematoxylin and eosin staining. Animals inoculated with PBS or PBS plus adjuvant were used as controls.

### ELISA and plaque reduction neutralization assay

Quantification of generated anti-DENV3 IgG antibodies was performed through an in-house ELISA, as previously described [45]. The induction of anti-DENV3 neutralizing antibodies was assessed by a PRNT_50_ test [33, 49] in LLC-MK2 cells (ATCC, USA). Sera were twofold serially diluted, beginning with a 1/20 dilution and up to 1:320, and used against a DENV-3 strain isolated from a human infection [45]. Each dilution was tested in duplicate and the number of plaque-forming units (PFU) was recorded as the average of the number observed in each test.

### Cellular proliferation assay

The induction of specific anti-DENV3 cellular responses was evaluated through splenocyte proliferation detection after ex vivo stimulation with DENV3E using the thymidine analog bromodeoxyuridine (BrdU) labeling kit (Millipore, Bethesda, USA). Spleen cells were plated in 96-well plates (2 × 10^5^ cells per well) in RPMI media supplemented with 10% fetal bovine serum. Mice splenocytes received 10 µg mL^−1^ of recombinant DENV3E as stimulus, while 2 µg mL^−1^ of concanavalin A (ConA) was added to the positive controls and RPMI to the negative controls. After initial incubation (72 h at 37 °C with 5% CO_2_), cells were further incubated for 18 h in the presence of BrdU. BrdU incorporation as an indication of cell proliferation was measured at 450 nm using an Asys Hitech Expert Plus spectrophotometer.

### Evaluation of cytokine production

The production of specific cytokines (IFN-γ, IL-10 and IL-6) in the supernatant of DENV3E stimulated splenocytes was measured using detection kits (R&D Systems^®^) and protocols recommended by the supplier. Results are expressed in pg of cytokine mL^−1^.

## Abbreviations

CYD-TDV: Sanofi Pausteur’s Dengvaxia^®^ vaccine
DENV: *Dengue virus*
CNT: carbon nanotubes
SWNT: single walled carbon nanotubes
MWNT: multi-walled carbon nanotubes
ƒ-CNT: functionalized carbon nanotubes
DNA: deoxyribonucleic acid
DENV3E: recombinant dengue virus 3 envelope protein
SDS-PAGE: sodium dodecyl sulfate polyacrylamide gel electrophoresis
TEM: transmission electron microscopy
AFM: atomic force microscopy
PBS: phosphate-buffered saline
ELISA: enzyme-linked immunosorbent assay
PRNT: plaque reduction neutralization test
PBMC: peripheral blood mononuclear cell
IFN: interferon
IL: interleukin
APC: antigen-presenting cell
MHC-1: major histocompatibility complex 1
Th: T helper cells
TLR: toll-like receptor
